# Hypophosphites in the Tooth-ache of Pregnancy

**Published:** 1869-06

**Authors:** 


HYPOPHOSPHITES IN THE TOOTH-ACHE OF PREGNANCY.—Dr. W. H. Sterling, of Burlington, N. J., (*American Medical Journal*) had a patient who was seized during pregnancy with severe tooth-ache, and rapid decay of the teeth. After the failure of other remedies, and acting on the idea that the "bone and nerve forming elements in her system were not sufficient for both fetus and mother," he prescribed the hypophosphites of lime, soda, potassa, and manganese, in two grain doses each, three times daily, in the form of glycerole. "The relief was immediate and permanent, the pain entirely removed, and the decay of her teeth was arrested, and her general health very much improved, with the renewal of her physical strength and mental vigor."

